# Serum galectin-9 levels are elevated in the patients with type 2 diabetes and chronic kidney disease

**DOI:** 10.1186/1471-2369-14-23

**Published:** 2013-01-22

**Authors:** Yuko Kurose, Jun Wada, Motoko Kanzaki, Sanae Teshigawara, Atsuko Nakatsuka, Kazutoshi Murakami, Kentaro Inoue, Takahiro Terami, Akihiro Katayama, Mayu Watanabe, Chigusa Higuchi, Jun Eguchi, Nobuyuki Miyatake, Hirofumi Makino

**Affiliations:** 1 Department of Medicine and Clinical Science, Okayama University Graduate School of Medicine, Dentistry and Pharmaceutical Sciences, Okayama, Japan; 2 Department of Hygiene, Faculty of Medicine, Kagawa University, Kagawa, Japan

**Keywords:** Type 2 diabetes, Glomerular filtration, Inflammation, Kidney disease, Nephropathy

## Abstract

**Background:**

Galectin-9 (Gal-9) induces apoptosis in activated T helper 1 (T_H_1) cells as a ligand for T cell immunoglobulin mucin-3 (Tim-3). Gal-9 also inhibits the G1 phase cell cycle arrest and hypertrophy in db/db mice, the hallmark of early diabetic nephropathy, by reversing the high glucose-induced up-regulation of cyclin dependent kinase inhibitors such as p27^Kip1^ and p21^Cip1^.

**Methods:**

We investigated the serum levels of Gal-9 in the patients with type 2 diabetes and various stages of chronic kidney disease (CKD) (n=182).

**Results:**

Serum Gal-9 levels in the patients with type 2 diabetes were 131.9 ± 105.4 pg/ml and Log_10_Gal-9 levels significantly and positively correlated with age (r=0.227, p=0.002), creatinine (r=0.175, p=0.018), urea nitrogen (r=0.162, p=0.028) and osmotic pressure (r=0.187, p=0.014) and negatively correlated with estimated glomerular filtration rate (eGFR) (r=−0.188, p=0.011). Log_10_Gal-9 levels increased along with the progression of GFR categories of G1 to G4, and they were statistically significant by Jonckheere-Terpstra test (p=0.012). Log_10_Gal-9 levels remained similar levels in albuminuria stages of A1 to A3.

**Conclusion:**

The elevation of serum Gal-9 in the patients with type 2 diabetes is closely linked to GFR and they may be related to the alteration of the immune response and inflammation of the patients with type 2 diabetes and CKD.

## Background

Galectins are β-galactoside binding protein and involved in various biological processes such as development, organogenesis, oncogenesis, cell adhesion, cell cycle regulation and immunity [1]. Mouse and rat galectin-9 (Gal-9) was identified [2,3] and its human homologue was independently cloned by using autoreactive antibodies in Hodgikin’s disease [4]. Galectin-9 exerted apoptotic potential against thymocytes [2], suggesting their important roles in the negative selection of thymocytes. Gal-9 lacking signal peptide is secreted out by non-classical pathway under inflammatory state and induced apoptosis in activated CD8^+^ T cells [5,6] and activated T helper 1 (T_H_1) cells [7], suggesting a potential mechanism to eliminate the activated T cells at termination of the immune response in inflammatory tissues. T cell immunoglobulin mucin-3 (Tim-3) has been identified as a receptor for Gal-9, Gal-9 induces apoptosis in CD4^+^Tim-3^+^ T_H_1 cells, and Gal-9-Tim-3 pathway negatively regulates T_H_1 immunity [7].

In addition to apoptotic potential to immune mediated cells, Gal-9 is a cell cycle regulator and it altered the status of cell proliferation and cell cycle arrest. In diabetic nephropathy, G1 phase cell cycle arrest and hypertrophy in mesangial and glomerular epithelial cells are the characteristic pathological change and up-regulation of cyclin dependent kinase inhibitors such as p27^Kip1^ and p21^Cip1^ are critically involved in this process. The injection of recombinant protein of Gal-9 into db/db mice inhibited the glomerular hypertrophy and albuminuria, and Gal-9 reversed up-regulation of p27^Kip1^ and p21^Cip1^ and promoted the progression of cell cycle from G1 phase in cultured mesangial cells [8].

The line of evidences led us to investigate the serum levels of Gal-9 in the patients with type 2 diabetes and various stages of chronic kidney disease (CKD), since the alteration of serum Gal-9 levels may influence the status of immune responses and cell cycle regulation in the various cells including kidney cells.

## Methods

### Patients

Japanese patients with type 2 diabetes (n=182, 60.4 ± 14.4 years) were enrolled into this study. The patients with type 2 diabetes were treated with oral hypoglycemic agents (n=132), insulin treatment (n=72) or both (n=32). The patients with eGFR < 15 ml/min/1.73 m^2^ or under dialysis were excluded from the current study. All recruited patients with type 2 diabetes agreed to measure serum Gal-9 levels. The study was conducted in accordance with the ethical principle of the Declaration of Helsinki and approved by ethical committee of Okayama University Graduate School of Medicine, Dentistry and Pharmaceutical Sciences. We obtained informed consent from each patient.

### Blood sampling and assays

We measured overnight fasting serum levels of total cholesterol and low density lipoprotein (LDL) cholesterol, high density lipoprotein (HDL) cholesterol, triglycerides (L Type Wako Triglyceride · H, Wako Chemical, Osaka), uric acid, serum creatinine (Cr), serum urea nitrogen (UN), plasma glucose, and HbA1c. Urinary albumin was measured in random spot urine samples by standard immuno-nephelometric assay. The urinary albumin-creatinine ratio (ACR) was calculated. Estimated glomerular filtration rate (eGFR) was calculated by equation; eGFR (ml/min/1.73 m^2^)=194 × Cr^-1.094^ × age^-0.287^ in male and eGFR (ml/min/1.73 m^2^)=194 × Cr^-1.094 ^× age^-0.287^ × 0.739 in female [9]. By using the definition and classification of chronic kidney disease [Kidney Disease: Improving Global Outcomes (KDIGO)] [10], all patients were classified into albuminuria and GFR category. In albuminuria stages, the patients were classified into three groups; A1 (<30 mg/gCr), A2 (30–299 mg/gCr) and A3 (≥300 mg/gCr). In GFR stages, they were classified into 4 groups; G1 (≥90 ml/min/1.73 m^2^), G2 (60–89 ml/min/1.73 m^2^), G3 (30–59 ml/min/1.73 m^2^), and G4 (15–29 ml/min/1.73 m^2^). Osmotic pressure was calculated by osmotic pressure (mOSM/l)=2×[Na (mmol/l) +K (mmol/l)]+[plasma glucose (mg/dl) / 18)+[UN (mg/dl) / 2.8]. Serum Gal-9 levels were measured with ELISA kit for human Gal-9 (Uscn, Wuhan, P.R. China). According to the manufacturer’s data sheet, sensitivity is less than 2.8 pg/ml and no significant cross-reactivity or interference among human Gal-9 and analogues was observed.

### Statistical analysis

All data are expressed as mean ± standard deviation (SD) values. Since serum Gal-9 concentrations did not show normal distribution, they were log-transformed and nonparametric tests were employed. Spearman correlation coefficients were used to evaluate whether serum Log_10_Gal-9 levels correlated with various parameters. To determine the variables independently associated with serum Gal-9 levels in the patients with type 2 diabetes, multiple regression analysis was performed by including age, osmotic pressure and eGFR as independent variables. Gal-9 levels and various clinical parameters in albuminuria and GFR stages were compared by Jonchheere-Terpstra test. Jonchheere-Terpstra test is similar to Kruskal-Wallis test but applied to samples with *a priori* ordering, *e.g.*, stages of disease. *P* values less than 0.05 were considered statistically significant. Statistical analysis was performed with PASW Statistics 18 (SPSS Inc., Chicago, IL).

## Results

### Serum Gal-9 levels correlated with age, Cr, UN, eGFR and osmotic pressure

**Figure 1:**
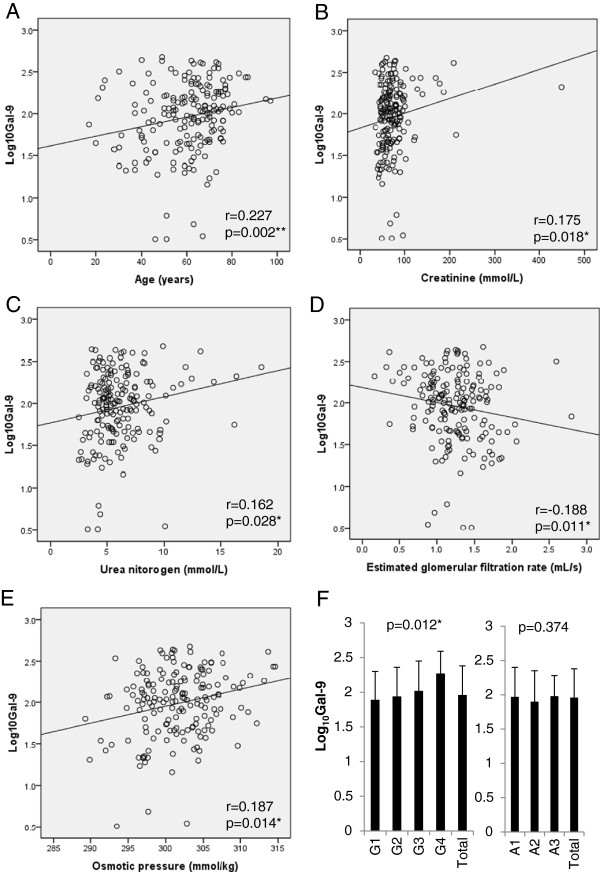
**Serum galectin-9 levels in the patients with type 2 diabetes (n=182). **The simple correlations of Log10Gal-9 and various parameters; age (**A**), serum creatinine (**B**), serum urea nitrogen (**C**), estimated glomerular filtration rate (eGFR) (**D**), and osmotic pressure (**E**). Log10Gal-9 levels are also shown in GFR stages and albuminuria stages and differences are analyzed by Jonckheere-Terpstra test (**F**).

Serum Gal-9 did not show correlation with blood glucose, HbA1c levels and variation of the treatments in type 2 diabetes. Serum Gal-9 levels in the patients with type 2 diabetes were 131.9 ± 105.4 pg/ml. Serum Gal-9 levels significantly and positively correlated with age (r=0.227, p=0.002), Cr (r=0.175, p=0.018), UN (r=0.162, p=0.028) and osmotic pressure (r=0.187, p=0.014) (Figure 1A-1C, 1E). Serum Gal-9 levels significantly and negatively correlated with eGFR (r=−0.188, p=0.011) (Figure 1D). The linear regression analyses were followed by a stepwise multiple regression analysis using serum Gal-9 levels as the dependent variables to further analyze the significant predictors (Table 1). Age, osmotic pressure and eGFR were used as independent variables. By stepwise analysis in model 1, only osmotic pressure independently correlated with serum Gal-9 levels. By including all variables demonstrating significant simple correlation with serum Gal-9 levels, only osmotic pressure significantly predicted the serum Gal-9 levels but other parameters did not enter the equation at significant levels in model 2 (Table 1).

**Table 1:** Multiple linear regression analysis in the patients with type 2 diabetes (n=182) using serum galectin-9 levels as dependent variables

**Model**	**Independent variables**	**Unstandardized coefficient**		**Standardized coefficients**	**t value**	**P value**	**Model r**^**2**^
		**B**	**Standard Error**	**Beta**			
1	Osmotic pressure (mmol/kg)	0.020	0.006	0.239	3.187	0.002	0.057
2	Age (year)	0.003	0.003	0.102	1.141	0.255	0.068
	Osmotic pressure (mmol/kg)	0.018	0.007	0.209	2.521	0.013	
	eGFR (mL/s)	−0.012	0.102	−0.011	−0.113	0.910	

Age, osmotic pressure and estimated glomerular filtration rate (eGFR) are used as independent variables in stepwise multiple linear regression analysis in model 1. In model 2, all parameters are included in the analysis.

### Serum Gal-9 levels elevated with the progression of GFR stages

Next, we investigated the serum Gal-9 levels in various stages of CKD. Age, ACR, Cr, UN, eGFR and osmotic pressure were significantly increased during the progression of GFR stages by Jonckheere-Terpastra test (Table 2). Log_10_Gal-9 levels also increased in the later stages of GFR and they were statistically significant by Jonchheere-Terpstra test (p=0.012) (Table 2 and Figure 1F). Since age differs significantly between G1 to G4 stages in Table 2, we selected the patients older than 60 years old (n=107) and performed Jonckheere-Terpastra analysis. Log_10_Gal-9 levels were still statistically different in G1 to G4 stages (p = 0.046). In albuminuria stages, ACR, Cr, UN, eGFR and osmotic pressure were significantly altered by Jonckheere-Terpstra (Table 3). However, Log_10_Gal-9 levels remained similar levels in A1 to A3 stages (Table 3 and Figure 1F).

**Table 2:** Comparison of various parameters in glomerular filtration stages of chronic kidney disease in type 2 diabetes patients (n=182)

	**G1**	**G2**	**G3**	**G4**	**Total**	**Jonckheere-Terpstra**
Number (male/female)	37 (21 / 16)	99 (37 / 62)	41 (12 / 29)	5 (1 / 4)	182 (71 / 111)	0.009**
Age (years)	50.0±15.5	60.7±10.8	70.2±11.7	73.8±7.8	60.4±14.4	6.43×10^-10^**
BMI (kg/m^2^)	27.0±6.9	25.5±4.8	24.7±4.0	25.9±1.7	25.6±5.1	0.242
SBP (mmHg)	128.9±14.7	129.7±15.4	129.6±18.3	128.2±18.4	129.5±15.9	0.939
DBP (mmHg)	77.7±10.8	74.5±10.9	73.1±12.9	67.4±9.8	74.7±11.4	0.031*
PG (mmol/L)	9.6±4.2	8.6±3.1	8.8±3.1	8.6±3.1	8.9±3.3	0.113
HbA1c (%)	7.90±1.54	7.25±1.01	7.13±0.78	7.04±0.88	7.35±1.12	0.029*
HbA1c (mmol/mol)	65.3±16.0	58.5±10.5	57.3±8.1	73.1±9.1	76.4±11.6	0.029*
ACR (mg/gCr)	19.6±27.7	130.6±344.4	421.4±1191	702.1±603.0	196.6±655.0	0.005**
Cr (μmol/L)	49.5±8.8	66.3±11.5	94.6±23.0	245.8±114.9	74.3±39.8	3.45×10^-21^**
UN (mmol/L)	4.7±1.6	5.7±1.6	20.6±7.4	15.7±2.1	6.1±2.5	1.60×10^-9^**
eGFR (mL/s)	1.76±0.26	1.25±0.14	0.85±0.13	0.33±0.10	1.24±0.37	8.80×10^-30^**
Osmotic pressure (mmol/kg)	299.3±4.0	301.2±4.3	303.0±4.2	312.9±1.2	301.6±4.8	4.78×10^-6^**
T-Cho (mmol/L)	4.77±0.81	4.93±0.88	4.80±1.30	5.05±0.90	4.87±0.97	0.736
TG (mmol/L)	1.51±0.74	1.51±0.77	1.68±0.91	2.19±1.16	1.56±0.81	0.391
HDL-C (mmol/L)	1.48±0.36	1.61±0.54	1.53±0.45	1.27±0.06	1.56±0.49	0.776
LDL-C (mmol/L)	2.67±0.70	2.79±0.68	2.73±0.80	3.09±0.99	2.75±0.72	0.753
Log_10_Gal-9 (pg/mL)	1.89±0.41	1.94±0.42	2.02±0.43	2.27±0.32	1.96±0.42	0.012*

BMI, body mass index; SBP, Systolic Blood Pressure; DPB, Diastolic Blood Pressure; PG, Plasma glucose; ACR, albumin / creatinine ratio; Cr, serum creatinine; UN, serum urea nitrogen; eGFR, estimated glomerular filtration ratio; T-Cho, Total cholesterol; TG, Triglyceride; HDL-C, HDL cholesterol; LDL-C, LDL cholesterol; Log_10_Gal-9, Log_10_galectin-9 (pg/mL). *, p < 0.05; **, p < 0.01.

**Table 3:** Comparison of various parameters in albuminuria stages of chronic kidney disease in type 2 diabetes patients (n=182)

	**A1**	**A2**	**A3**	**Total**	**Jonckheere-Terpstra**
Number (male/female)	108 (46 / 62)	47 (17 / 30)	27 (8 / 19)	182 (71 / 111)	0.075
Age (years)	60.0±14.0	63.5±13.5	62.0±12.7	60.4±14.4	0.199
BMI (kg/m^2^)	25.3±1.5	26.2±6.2	27.3±5.1	25.6±5.1	0.227
SBP (mmHg)	128.8±13.3	132.7±19.7	137.0±20.0	129.5±15.9	0.071
DBP (mmHg)	75.1±9.9	72.7±13.1	78.3±16.6	74.7±11.4	0.982
PG (mmol/L)	8.5±3.0	9.6±3.9	9.1±2.7	8.9±3.3	0.170
HbA1c (%)	7.27±0.87	7.51±1.35	7.61±1.41	7.35±1.12	0.611
HbA1c (mmol/mol)	75.5±9.0	78.0±14.0	79.0±14.6	76.3±11.6	0.611
ACR (mg/gCr)	11.2±6.2	110.7±83.4	1474±1444	196.6±655.0	5.06×10^-23^**
Cr (μmol/L)	69.0±40.7	73.4±19.4	114.9±53.9	74.3±39.8	2.44×10^-4^**
UN (mmol/L)	5.8±2.1	6.5±1.9	9.1±4.1	6.1±2.5	2.04×10^-4^**
eGFR (mL/s)	1.29±0.34	1.17±0.30	0.86±0.37	1.24±0.37	2.55×10^-4^**
Osmotic pressure (mmol/kg)	301.0±4.0	300.9±4.7	306.7±4.8	301.6±4.8	0.013*
T-Cho (mmol/L)	4.85±0.92	5.00±1.17	4.98±0.94	4.87±0.97	0.688
TG (mmol/L)	1.44±0.71	1.75±0.82	1.72±0.84	1.56±0.81	0.051
HDL-C (mmol/L)	1.57±0.49	1.52±0.51	1.56±0.54	1.56±0.49	0.413
LDL-C (mmol/L)	2.77±0.62	2.90±0.85	2.78±0.77	2.75±0.72	0.890
Log_10_Gal-9 (pg/mL)	1.97±0.43	1.90±0.45	1.98±0.30	1.96±0.42	0.374

BMI, body mass index; SBP, Systolic Blood Pressure; DPB, Diastolic Blood Pressure; PG, Plasma glucose; ACR, albumin / creatinine ratio; Cr, serum creatinine; UN, serum urea nitrogen; eGFR, estimated glomerular filtration ratio; T-Cho, Total cholesterol; TG, Triglyceride; HDL-C, HDL cholesterol; LDL-C, LDL cholesterol; Log_10_Gal-9, Log_10_galectin-9 (pg/mL). *, p < 0.05; **, p < 0.01.

## Discussion

The presence of galectin-9 in human serum was well-documented in previous reports. Serum galectin-9 was elevated in hepatitis C infection and it was released from Kupffer cells in the liver [11]. In addition, oral administrations of dietary synbiotic bacteria such as *Bifidobacterium breve* M-16V increased the expression of galectin-9 in intestinal epithelial cells, increased serum galectin-9 levels, and prevented allergic responses in human [12]. Galectin-9 is also stimulated and released from various cells by interferon-γ in human endothelial cells [13], fibroblasts [14], pancreatic β cells [15], and Kupffer cells [11]. Galectin-9 is vulnerable to digestion by proteolytic degradation; however, it was reported that galectin-9 is inserted into exosome and released, thus it is protected by enzymatic degradation, and the intact 36 kDa molecule was demonstrated in the serum exosome fraction [16]. Galectin-9 is also abundantly expressed in the cytoplasm of tubular cells and kidney may contribute the circulating Gal-9; however, regulation of the release of Gal-9 from kidney cells is completely unknown [2,3].

In current clinical investigation, simple correlation of Log_10_Gal-9 levels with age, Cr, UN, and eGFR suggested that serum Gal-9 levels closely related to the renal function in patients with type 2 diabetes. The molecular weight of Gal-9 is ~36 kDa and it would be filtered through glomerular capillaries and the reduction of GFR may be linked to the elevation of serum Gal-9 levels. Actually, log_10_Gal-9 levels increased along with the progression of GFR stages, *i.e.* G1 to G4. In diabetic kidney disease, albuminuria also increased during the progression of the disease and Gal-9 may be actively filtered through glomerular basement membranes; however, serum Gal-9 levels did not negatively correlate with urinary albumin excretion and serum Gal-9 levels were not altered in the progression of albuminuria stages from A1 to A3. Although both of the reduced filtration of Gal-9 and loss of Gal-9 into the urine may be the determinants for the serum Gal-9 levels, the current clinical study suggested that GFR mainly defined the serum Gal-9 levels.

In addition to GFR, serum Gal-9 levels also revealed simple correlation with osmotic pressure. Since serum osmotic pressure is determined by the concentrations of sodium, potassium, plasma glucose and UN, the osmotic pressure in the patients of type 2 diabetes would be elevated by the impairment of renal function or by hyperglycemia. Multiple linear regression analysis revealed that osmotic pressure is only significant predictor for serum Gal-9 levels by employing age, osmotic pressure and eGFR as independent variables. A novel model for non-classical secretion of fibroblast growth factor 1 (FGF1), FGF2, and galectins without signal peptide have been reported, namely oncotic release, where a change in the colloidal osmotic pressure by serum deprivation in the culture cells creates the nonlethal oncotic pores in the plasma membranes through which proteins are released [17]. There are no reports whether the increase in osmotic pressure alters the plasma membrane and it stimulates the secretion of Gal-9 via non-classical pathway; however, the current study suggested that osmotic pressure might be the stimulator for the release of Gal-9 and the future studies are required to support this evidence.

Since the current investigation is cross-sectional clinical study, it is difficult to conclude whether elevated serum Gal-9 levels are protective or promoting for the progression of diabetic nephropathy. Gal-9 induces apoptosis in CD4^+^Tim-3^+^ T_H_1 cells, and Gal-9-Tim-3 pathway negatively regulates T_H_1 immunity, thus the elevation of serum Gal-9 may be beneficial in the progression of diabetic nephropathy by negatively regulating the immune responses and inflammation [18]. In addition, the elevation of serum Gal-9 concentrations may inhibit the G1 cell cycle arrest and hypertrophy of the kidney cells [8]. Thus, the follow-up cohort study may be required to clarify whether the elevated serum Gal-9 levels in type 2 diabetes are preventive for the progression of diabetic nephropathy. In recent series of the investigations, Gal-9 is also reported to regulate the virus specific T-cell response [19], T cell immunity in hepatitis C infection [11], anti-microbial immunity [20], it is an important clinical question whether elevated serum levels of Gal-9 in the patients with type 2 diabetes and diabetic nephropathy are related to the susceptibility for various infection in the future studies.

## Conclusion

Serum Gal-9 levels in the patients with type 2 diabetes significantly and positively correlated with age, creatinine, urea nitrogen and osmotic pressure and negatively correlated with estimated glomerular filtration rate (eGFR). Log_10_Gal-9 levels increased along with the progression of GFR categories of G1 to G4, and they were statistically significant by Jonckheere-Terpstra test (p=0.012). The elevation of serum Gal-9 in the patients with type 2 diabetes is closely linked to GFR and they may be related to the alteration of the immune response and inflammation of the patients with type 2 diabetes and CKD.

## Competing interests

The authors declare that they have no competing interests.

## Authors’ contributions

YK, JW, AN, ST, AN and HM participated in the design of the study and KZ, KI, TT, AK, MW, and CH participated in the recruitment of the patients. YK, MK and ST carried out ELISA measurements of all samples. YK, JW, JE, NM and HM conceived of the study, participated in coordination, performed the statistical analyses and helped to draft the manuscript. All authors read and approved the final manuscript.

## Pre-publication history

The pre-publication history for this paper can be accessed here:

http://www.biomedcentral.com/1471-2369/14/23/prepub
